# Proteoglycan 4 downregulation in a sheep meniscectomy model of early osteoarthritis

**DOI:** 10.1186/ar1898

**Published:** 2006-01-31

**Authors:** Allan A Young, Susan McLennan, Margaret M Smith, Susan M Smith, Martin A Cake, Richard A Read, James Melrose, David H Sonnabend, Carl R Flannery, Christopher B Little

**Affiliations:** 1 Raymond Purves Research Laboratory, Institute of Bone and Joint Research, Royal North Shore Hospital, University of Sydney, Pacific Highway, St Leonards, NSW 2065, Australia; 2 Department of Endocrinology, Royal Prince Alfred Hospital and Department of Medicine, University of Sydney, Missenden Road, Camperdown, NSW 2050, Australia; 3 School of Veterinary and Biomedical Sciences, Murdoch University, South Street, Murdoch, WA 6150, Australia; 4 Wyeth Research, 200 Cambridge Park Drive, Cambridge, MA 02140, USA

## Abstract

Osteoarthritis is a disease of multifactorial aetiology characterised by progressive breakdown of articular cartilage. In the early stages of the disease, changes become apparent in the superficial zone of articular cartilage, including fibrillation and fissuring. Normally, a monolayer of lubricating molecules is adsorbed on the surface of cartilage and contributes to the minimal friction and wear properties of synovial joints. Proteoglycan 4 is the lubricating glycoprotein believed to be primarily responsible for this boundary lubrication. Here we have used an established ovine meniscectomy model of osteoarthritis, in which typical degenerative changes are observed in the operated knee joints at three months after surgery, to evaluate alterations in proteoglycan 4 expression and localisation in the early phases of the disease. In normal control joints, proteoglycan 4 was immunolocalised in the superficial zone of cartilage, particularly in those regions of the knee joint covered by a meniscus. After the onset of early osteoarthritis, we demonstrated a loss of cellular proteoglycan 4 immunostaining in degenerative articular cartilage, accompanied by a significant (*p *< 0.01) decrease in corresponding mRNA levels. Early loss of proteoglycan 4 from the cartilage surface in association with a decrease in its expression by superficial-zone chondrocytes might have a role in the pathogenesis of osteoarthritis.

## Introduction

Proteoglycan 4 (PRG4), which is homologous to lubricin [1], superficial zone protein (SZP) [2], megakaryocyte-stimulating factor precursor [2] and camptodactyly-arthropathy-coxavara-pericarditis protein [3], is a lubricating glycoprotein believed to be primarily responsible for boundary lubrication in synovial joints [4]. As previously suggested [5], we also refer to these molecules with a common immunoreactivity as PRG4 in the present study. PRG4 is a component of synovial fluid and is synthesised by the superficial chondrocytes in both normal articular cartilage and synovial cells [6]. A thin layer of PRG4 is present at the surface of normal articular cartilage; however, the relative contributions of synthesis from superficial chondrocytes and from synovial cells to the formation of this layer remains to be established [7].

Articular cartilage demonstrates zonal variation in both composition and structural arrangement of the extracellular matrix, reflecting its functional role [8]. The morphology of the chondrocytes also differs with depth from the surface, assuming a more flattened appearance in the superficial zone and aligning parallel to the articulating surface [8]. Alterations in the superficial zone are known to occur early in osteoarthritis (OA), a progressive and debilitating disease characterised by degeneration and loss of articular cartilage. Proteolytic degradation of the extracellular matrix and alterations in resident chondrocyte synthetic activity results in disruption of the structural integrity of articular cartilage. Increased apoptosis, or programmed cell death, is also observed in OA and to a greater extent in the superficial zone(s) [9].

Deficiency of PRG4 results in a loss of the chondroprotection normally provided to articulating surfaces; it has therefore been implicated in the pathogenesis of OA [10,11]. PRG4 has been shown to still be present in late-stage human OA [7]; however, little is known about the turnover of PRG4 during the early stages of the disease process. The aim of the present study was therefore to determine the changes in cartilage PRG4 expression and immunolocalisation occurring early in the pathogenesis of OA with the use of an established animal model. We also sought to evaluate regional patterns of PRG4 expression and localisation across the ovine knee joint.

## Materials and methods

### Animal model

Twelve four-year-old female pure-bred Merino sheep were used for this study. Six of the sheep underwent open lateral meniscectomy of both stifle (knee) joints as described previously [12], and the other six underwent a sham operation identical in all aspects except that the lateral meniscus was not excised. After recovery from surgery, the animals were maintained in an open paddock for three months before being killed. The protocol used for this study was approved by the animal ethics committee of Murdoch University (AEC 832R/00).

### Tissue preparation and histology

**Figure 1:**
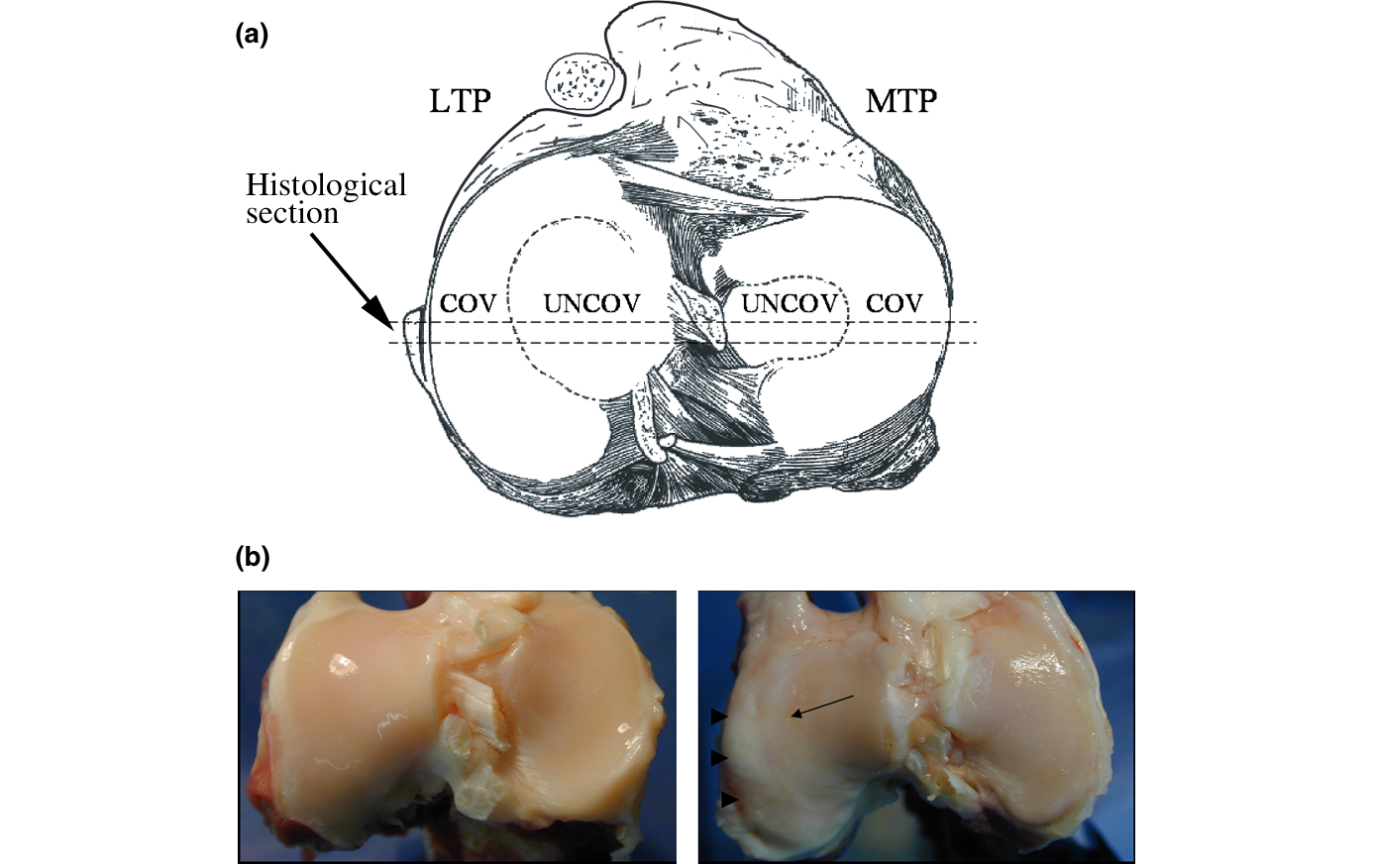
Sheep meniscectomy model of early osteoarthritis **(a) **Schematic representation of left ovine tibial plateau demonstrating medial (MTP) and lateral (LTP) plateaux, further separated into regions normally covered (COV) and uncovered (UNCOV) by menisci. Full-thickness coronal sections were taken for histological evaluation as indicated by the broken line. **(b) **Photographs demonstrating typical osteoarthritic changes observed after lateral meniscectomy (MEN), including surface fibrillation (arrow) and osteophyte formation (arrowheads).

Full-depth articular cartilage was harvested from the medial tibial plateau (MTP) and lateral tibial plateau (LTP) in regions normally covered (COV) and uncovered (UNCOV) by menisci from either the right or left stifle joint, randomly selected (Figure 1a). Care was taken not to sample tissue from the joint margins or osteophytes. Tissue samples were snap frozen in liquid nitrogen before storage at -80°C until required. Coronal full-thickness osteochondral slabs (5 mm) were prepared through the mid weight-bearing region of the tibial plateau from the contralateral joint of each animal (Figure 1a). The specimens were then fixed, decalcified and processed before staining with toluidine blue and fast green as described previously [12]. Histological slides were subsequently evaluated by two independent observers with a modified Mankin scoring scheme, previously developed in our laboratory for this ovine model [12]. The modified Mankin score has a range of 0 to 29, the value increasing with severity of cartilage degeneration. In each compartment the worst score evident for the region examined was used to calculate the mean score (*n *= 6 for each group).

### Immunohistochemistry

To avoid the necessity for decalcification, articular cartilage spanning the entire MTP or LTP was micro-dissected as a single piece from the underlying subchondral bone after formalin fixation of osteochondral slabs of two representative sham-operated and meniscectomised animals, and these full-thickness cartilage specimens were then embedded in paraffin and 4 μm sections were deparaffinised in xylene and graded ethanols. Sections were digested with Proteinase K (code no. S3020; DakoCytomation, Glostrup, Denmark) for six minutes at room temperature followed by incubation in Protein Block Serum Free (code no. X909; DakoCytomation) for ten minutes at room temperature. Primary antibody incubations were performed overnight at 4°C with a 1:600 dilution of 06A10, a protein A purified rabbit polyclonal anti-PRG4 antibody generated by immunisation with a truncated form of recombinant human PRG4 (generously provided by Wyeth Pharmaceuticals, Boston, MA, USA). This antibody has previously been used to specifically detect PRG4 in the superficial zone of bovine articular cartilage [13]. Secondary antibody incubation and colour development were performed as described previously. The intensity and number of positively stained cells were evaluated across the width of the MTP and LTP by two observers [14].

### RNA extraction, reverse transcription and real-time quantitative PCR

About 100 mg of frozen cartilage samples was fragmented in a Mikro-Dismembrator (Braun Biotech International, Melsungen, Germany). Total RNA was isolated with the RNeasy Mini Kit (Qiagen, Valencia, USA) and quantified with a fluorimeter (Perkin Elmer, Beaconsfield, UK) with the use of SYBR^® ^Green II colour reagent (Cambrex Bio Science, Rockland, USA). The quality and integrity of total RNA were assessed on 2% (w/v) agarose gels stained with ethidium bromide. For all samples total RNA (1 μg) was reverse transcribed into cDNA with the Omniscript kit (Qiagen, Hilden, Germany) in accordance with the manufacturer's instructions. Real-time PCR was performed with a Prism 7000 Sequence Detection System from Applied BioSciences (Foster City, CA, USA). Primers were designed to bovine PRG4 (ovine sequence 99% homologous) (forward, 5'-CTGCCCAACATCAGAAAACCC-3' ; reverse, 5'-TTCCTTCGCCCATCAGTCTAAG-3') (Genbank accession no. AF056218) and generated a single PCR product in sheep, which was confirmed by sequencing (SUPAMAC, Sydney, Australia). cDNA (1 μl, corresponding to the reverse transcription of 25 ng of total RNA), 0.5 μl of each primer (10 μM), 0.5 μl of SYBR green (Molecular Probes, Sydney, Australia) and the Platinum Plus Taq (7 μl; Invitrogen, Sydney, Australia), with ROX (6-carboxy-X-rhodamine) (0.1 μl) used as an internal control. The thermal profile was as follows: 50°C for five minutes, 95°C for five minutes, and 40 cycles of 95°C for 15 seconds, 58°C for 30 seconds and 72°C for 30 seconds. Melt curves were also determined to demonstrate the specificity of the amplification. Standard curves were generated from plasmids (pGEM Teasy; Promega, Sydney, Australia) containing the PCR products, and the linear amplification range for both plasmid DNA and sample cDNA was determined. Analysis of the curves showed that the sample diluted in a parallel manner to the plasmid and that the cycle threshold (Ct) for all unknown samples fell within the linear range, allowing quantification of gene copy number relative to the plasmid concentration. Samples were analysed in triplicate and values were normalised to total RNA as recommended for experiments *in vivo *involving tissue specimens [15].

### Statistical analysis

Comparisons of non-parametric data from the modified Mankin histological scoring of the stained tissue sections were assessed with the Mann-Whitney *U *test. Statistical evaluation of significant differences in expression levels was undertaken with the unpaired Student *t *test with Benjamini-Hochberg correction for multiple comparisons.

## Results

### Gross morphology and histology

Lateral meniscectomy resulted in macroscopic joint changes characteristic of OA with cartilage fibrillation and erosion, particularly in the lateral compartment (Figure 1b). The most severe lesions were confined to the COV region of the LTP, with surface fibrillation and variable loss of the characteristic superficial zone cells. Histological grading of the cartilage specimens confirmed and quantified the regional histological observations. Modified Mankin scoring was significantly increased in the LTP COV (4.4 ± 1.2 to 17.6 ± 2.7; mean ± SD; *p *< 0.01) and LTP UNCOV (3.1 ± 1.1 to 6.7 ± 1.9; *p *< 0.01) regions after lateral meniscectomy; however, it remained unchanged in MTP COV (2.3 ± 0.9 to 4.3 ± 2.6) and MTP UNCOV (2.6 ± 1.2 to 4.3 ± 1.6) regions.

### Immunohistochemistry

**Figure 2:**
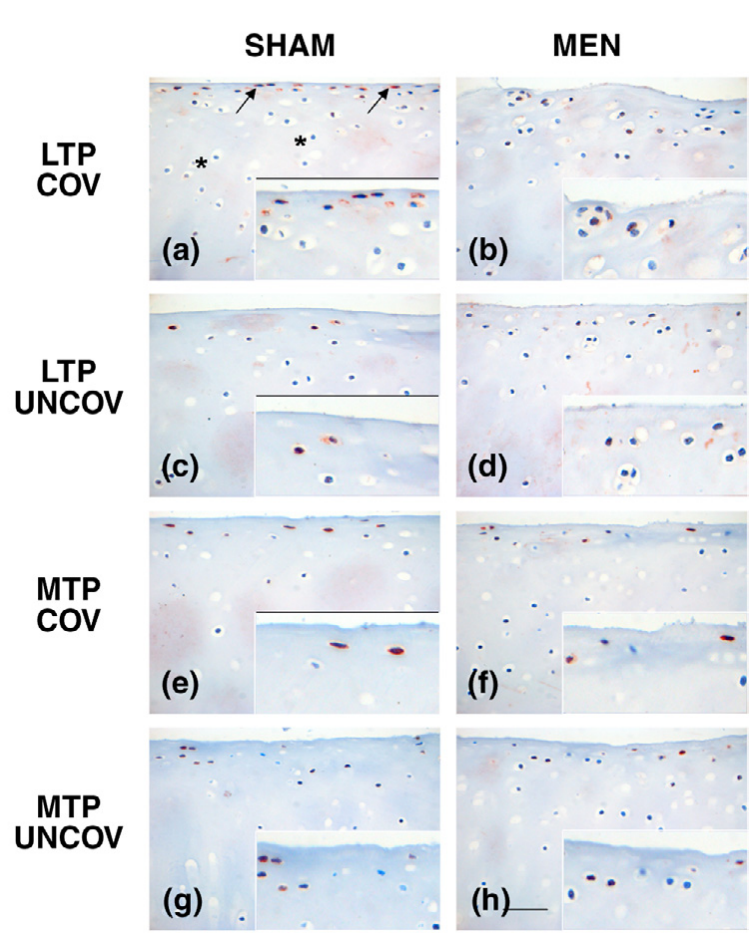
Regional immunolocalisation of proteoglycan 4 (PRG4) with polyclonal antibody 06A10 in ovine tibial plateau cartilage. Lateral and medial tibial plateaux (LTP and MTP, respectively) from regions covered (COV) or uncovered (UNCOV) by the meniscus in sham-operated and meniscectomised (MEN) joints are shown, including enlarged views of respective surface zones as insets. Representative PRG4-positive (arrows) and PRG4-negative (asterisks) chondrocytes are indicated. Scale bar, 150 μm.

PRG4 was immunolocalised to chondrocytes in the superficial zone of normal cartilage (Figure 2a,c,e,g; positive PRG4 indicated by red-brown colour) and little or no extracellular matrix staining was detected. Additionally, positive immunostaining cells were more prominent in the COV regions of normal cartilage than in the UNCOV regions. After meniscectomy, extensive loss of PRG4-positive cells was observed in the superficial zone of LTP specimens, both COV and UNCOV regions (Figure 2b,d), corresponding to areas of degenerative change. No obvious change in PRG4 immunostaining was observed in the MTP COV and UNCOV cartilage regions after meniscectomy (Figure 2f,h).

### Real-time quantitative PCR

**Figure 3:**
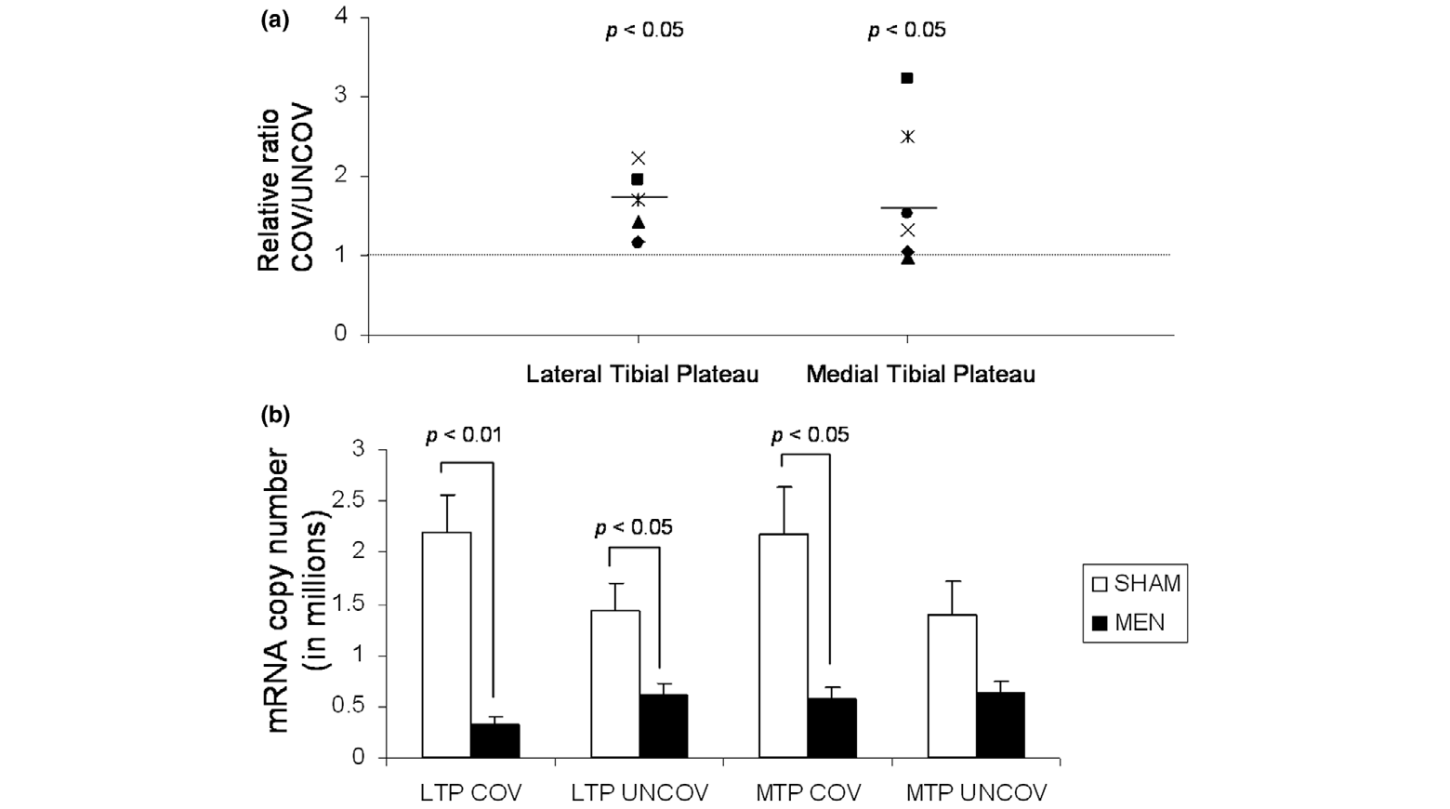
Quantitative real-time PCR. **(a) **Topographical variation in proteoglycan 4 (PRG4) expression in sham-operated ('normal') joints as demonstrated by the ratio of mRNA copy numbers from cartilage normally covered (COV) versus cartilage normally uncovered (UNCOV) by menisci; in each case the average value is indicated by the line (*n *= 6). The dotted horizontal line represents the expected ratio of 1 if there were no difference between regions. **(b) **PRG4 mRNA copy numbers (means ± SEM) from ovine articular cartilage after lateral meniscectomy (MEN). LTP, lateral tibial plateau; MTP, medial tibial plateau.

To evaluate whether topographical variation existed in the expression of PRG4 in normal joints, the ratio of mRNA levels in the COV versus UNCOV cartilage regions in sham-operated sheep was evaluated. PRG4 expression was found to be increased (1.8-fold, *p *< 0.05) in COV compared with UNCOV cartilage in the MTP and similarly increased (1.6-fold, *p *< 0.05) in COV compared with UNCOV cartilage in the LTP (Figure 3a).

After lateral meniscectomy, PRG4 mRNA levels were found to be significantly decreased compared with sham-operated controls in cartilage from the covered region of the LTP (7.0 fold, *p *< 0.01) (Figure 3b). PRG4 expression was also found to be decreased in the UNCOV region of the LTP (2.4 fold, *p *< 0.05) and in the COV region of the MTP (3.8 fold, *p *< 0.05).

## Discussion

To our knowledge this is the first report describing the immunolocalisation and expression of PRG4 in cartilage in an animal model of OA. Similarly to previous reports of PRG4 immunolocalisation in normal cartilage [5], in the present study we observed PRG4-positive cells typically in the superficial zone and not in the middle and deep zones. The lack of significant PRG4 staining in the superficial cartilage matrix in comparison with previous studies [13] may be related to tissue processing. The tibial plateaux cartilage underwent biomechanical testing before processing for histology. Physical removal of the surface PRG4 might have occurred during the 15-minute indentation testing, which requires repeated saline lavage and swabbing of the cartilage surface. After ovine lateral meniscectomy, there was a decrease in PRG4 immunostaining with a marked loss of PRG4-positive superficial zone chondrocytes in the degenerative cartilage of the lateral compartment. Importantly, this was not associated with an appearance of PRG4-positive cells in the middle or deep zones of cartilage. Previous studies performed in late-stage human OA cartilage collected at joint replacement surgery have reported the extension of PRG4-positive chondrocytes into the deeper zones, suggesting potential adaptive responses with disease progression [7] that were not apparent in the early stages of OA pathogenesis represented in the present study.

After ovine lateral meniscectomy, the most marked decrease in PRG4 expression was observed in the lateral compartment with the most severe histopathological alterations. However, mRNA levels were decreased in cartilage across the knee joint after meniscectomy regardless of associated degenerative changes. Darling and colleagues [16] recently demonstrated a threefold relative abundance of PRG4 mRNA levels in the superficial zone of normal articular cartilage. Decreases in the presence and/or viability of the superficial zone cells occurring early in the present model of OA therefore probably contributed to the observed decrease in cartilage PRG4 expression. Additionally, modulation of the chondrocyte phenotype in meniscectomised cartilage by mechanical or humoral factors were likely to have been associated with the downregulation in PRG4 expression observed in the compartments not undergoing active degeneration.

Topographical variation in PRG4 expression was observed in normal ovine knee joints in the present study, with increased expression in cartilage from regions protected by a meniscus, which was consistent with the immunolocalisation of PRG4 protein. Although a recent report [16] found no variation in PRG4 expression across distal femoral cartilage, we have previously demonstrated that topographical differences in chondrocyte metabolism were most pronounced in the tibial plateau [17]. These differences in cartilage metabolism in the tibial plateau were probably associated with the presence of the meniscus and its effect on mechanical loading of the cartilage. In the present study we postulate that not only variation in compressive mechanical loads but also potential shearing between the meniscus and underlying cartilage may modulate PRG4 expression in this region. Wong and colleagues [18] have previously demonstrated in chondrocyte-seeded alginate constructs that cyclic shear loading significantly upregulated PRG4 expression, whereas cyclic hydrostatic pressure was associated with a slight downregulation. Alterations in shear stress after lateral meniscectomy might therefore have contributed to the decreased cartilage PRG4 expression observed in the present study.

The mechanisms involved in regulating PRG4 expression and synthesis remain largely unknown. Increased catabolic pathways are present in OA, and the inflammatory cytokine IL-1 seems to be one of the most influential factors, demonstrating deleterious effects for cartilage *in vitro *and *in vivo*, acting to inhibit proteoglycan synthesis while promoting degradation of matrix components through both activation of proteases and stimulation of their production [19-23]. PRG4 seems to be highly regulated by IL-1, which has been shown to inhibit its secretion *in vitro*, and therefore potentially contributing to the pathogenesis of OA [2,24]. Conversely, it has shown that transforming growth factor-β (TGF-β) stimulates PRG4 synthesis and may be beneficial for normal cartilage function [2,24]. TGF-β has a significant role in promoting the anabolic activity of chondrocytes, and its expression has been reported to increase in early OA [25,26].

## Conclusion

Loss of PRG4, whether by altered synthesis or subsequent degradation, is likely to influence the functional properties of synovial joints. A focal decrease in PRG4 in early OA could have a role in the pathogenesis of cartilage degeneration. In the present study we have demonstrated in an animal model that early degeneration of cartilage was associated with the loss of PRG4 from articular cartilage concomitant with a significant decrease in its expression by chondrocytes in the superficial zone. Modulation of PRG4 in OA joints therefore provides a new approach to understanding the mechanisms of disease initiation and progression and offers potential as a novel therapeutic target for the treatment of this disorder.

## Abbreviations

COV = normally covered; IL = interleukin; LTP = lateral tibial plateau; MTP = medial tibial plateau; OA = osteoarthritis; PCR = polymerase chain reaction; PRG4 = proteoglycan 4; TGF = transforming growth factor; UNCOV = normally uncovered.

## Competing interests

The authors declare that they have no competing interests.

## Authors' contributions

AY designed the study, performed animal surgery, performed PCR experiments and drafted the manuscript. SM performed PCR experiments and helped draft the manuscript. MS designed primers for PCR experiments, performed histopathological cartilage scoring and helped draft the manuscript. SS performed histological and immunohistological preparations and helped draft the manuscript. MC performed animal surgery and helped draft the manuscript. RR performed animal surgery and helped draft the manuscript. JM assisted immunohistological preparations and helped draft the manuscript. DS was involved in the conception and design of the study and in the interpretation of the data, and critically revised the manuscript for important intellectual content. CF critically revised the manuscript for important intellectual content. CL performed histopathological cartilage scoring, analysed and interpreted the data and critically revised the manuscript for important intellectual content. All authors read and approved the final manuscript.
